# Experimental investigations of a manually versus an electrically driven skull drill for bedside usage

**DOI:** 10.1371/journal.pone.0215171

**Published:** 2019-04-18

**Authors:** Anne Carolus, Wolfgang Richter, Claus-Peter Fritzen, Kirsten Schmieder, Christopher Brenke

**Affiliations:** 1 Department of Neurosurgery, University Hospital Knappschaftskrankenhaus Bochum, Ruhr-University Bochum, Bochum, Germany; 2 Department of Mechanical Engineering, University of Siegen, Siegen, Germany; Mayo Clinic Minnesota, UNITED STATES

## Abstract

**Background:**

Manual skull drilling is an old but in modern neurosurgery still established procedure which can be applied quickly and universally in emergency situations. Electrical drilling requires more complex equipment and is usually reserved to the Operating Room (OR). It also seems desirable to apply an electrical drill for bedside usage but a suitable product does not exist so far.

**Method:**

Our experimental study using a manually and an electrically driven skull drill included a total of 40 holes drilled into synthetic biomechanical sheets. Half of the holes were produced with a prototype electrical drilling machine of the company Kaiser Technology and half of them with a traditional manual drill. Different drilling parameters such as the geometry of the borehole, the drilling forces and the drilling vibrations were captured during all experiments.

**Results:**

The electrical drilling needed higher vertical force by the operators and a longer time to penetrate the sheet. A reason was the relatively lower rotational speed provided by this particular drill. When drilling electrically the vibrations were substantially less which in turn led to a more precise shape of the holes (revealed by observation via a microscope).

**Conclusions:**

The electrification of bedside drilling can in principle enable emergency craniostomies to be performed with greater ease and accuracy. The power of the electric drive, however, must be at least equivalent to the power of the traditional manual drill. Otherwise, the vertical forces exerted on the scull by the operator become inhibitive. The challenge is to combine cost-efficiency and re-sterilizability of an electrically driven drilling machine which at the same time is small and simple enough to qualify for emergency applications.

## Introduction

Skull drilling is a surgical procedure that has been practiced since prehistoric times [1, 2, 3, 4]. In neurosurgery, the manual drilling technique survived to date [5, 6, 7]. Electrically driven drills are suitable for the Operating Room (OR) since they either depend on a drilling shaft or are large battery drills [8]. The manual twist drill prevailed in emergency situations, first and foremost for external ventricular drainage (EVD) application, because of several advantages: It is cheap, re-sterilizable, rugged (concerning mechanical loads and environmental conditions), totally independent from the OR with its electrical power supply [8] and has a compact size. On the contrary, the main problem of manual drilling is that the skull may be penetrated in an uncontrolled and abrupted way [9, 10, 11]. This may result in brain injury and intracerebral or acute epidural hematoma [10, 12, 13]. Some technical modifications to reduce these risks have been developed, for instance a pre-adjustable distance holder [1] or a percutaneous needle which replaces the turning drilling bit [14, 15]. But none of those devices has become widely accepted. Prediction and control of thrust forces and torques as well as possibly an optimized drill design are typical tasks related to mechanical engineering; see e.g. the publications [16, 17, 18]. This contribution tries to bridge the gap between engineering work and every day´s neurosurgical practice. Our hypothesis is that electrical drilling leads to significant advantages compared to manual drilling and that a well designed electrical drilling machine should be able to replace the twist drill in modern neurosurgery even in a bedside setting. The results of this study are thought to contribute to an improved list of specifications for an advanced electric drill, based on sound experimental results utilizing a battery-driven prototype drilling machine which was developed for bedside usage by Kaiser Technology, and a traditional manual drill. The study was carried out in cooperation of the Department of Neurosurgery, Knappschaftskrankenhaus Bochum and the Chair for Applied Mechanics, Department Mechanical Engineering at the University of Siegen.

## Material and methods

### Experimental setup

#### Bone sheets

**Fig 1:**
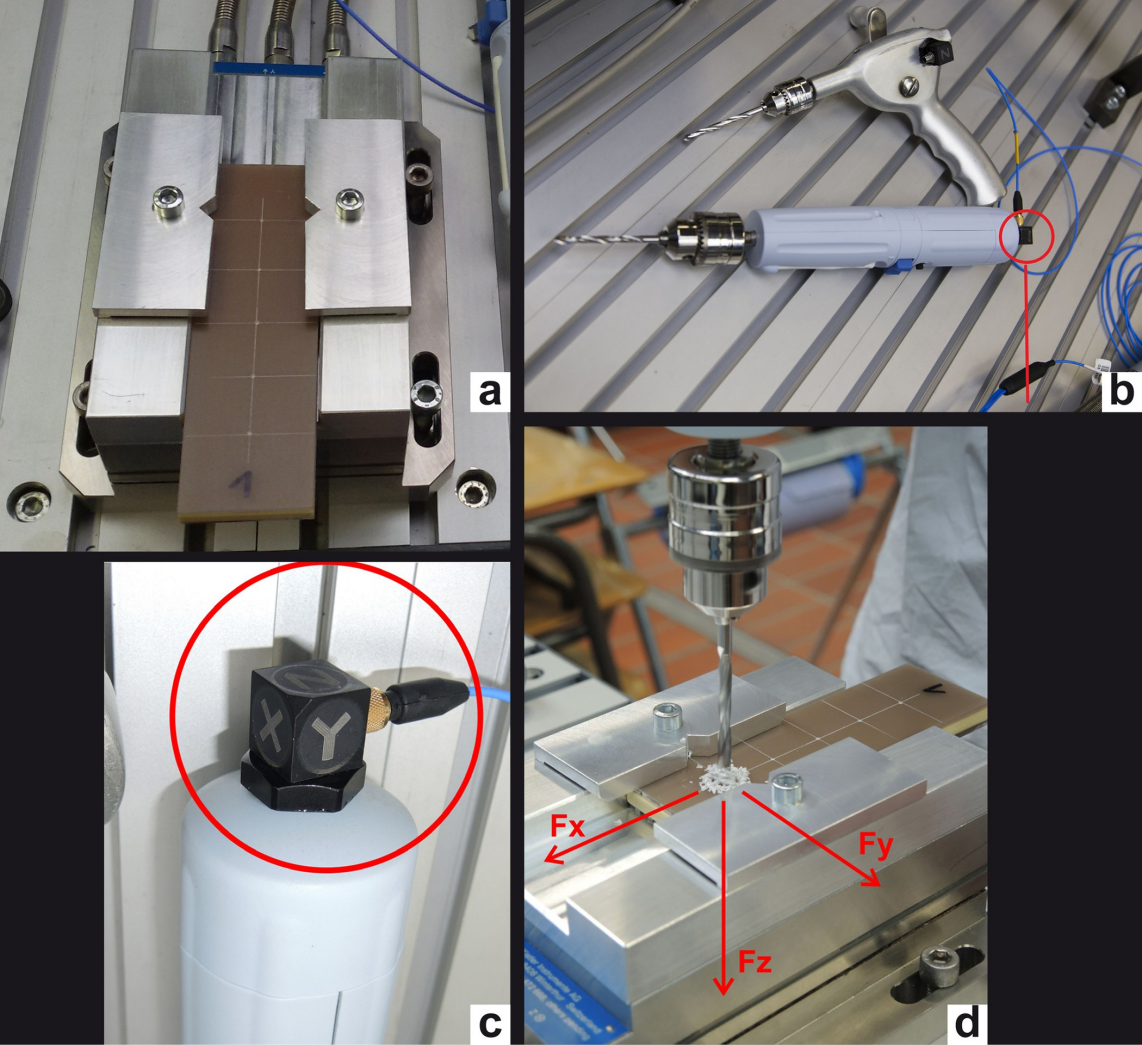
Experimental setup. (A) Test specimen clamped on load cell (B) Manual and electric drilling machine (C) triaxial acceleration sensor attached to the drilling machine (D) Force components resolved.

Experiments were performed utilizing synthetical biomechanical test specimens having structural properties comparable to a human skull bone. Initially, a neurosurgeon evaluated different designs and combinations in order to obtain a test specimen having structural properties as close as possible to a human skull bone. Each specimen is a combination of different materials provided by Sawbone. Two layers of 2 mm short fiber filled epoxy sheets and in between a 5 mm solid rigid polyurethane were glued together, resulting in 8 bicortical blocks with a size of 130 x 40 x 9.5 mm. Each specimen accommodated 5 bore holes with a distance of 20 mm to each other as well as to the edges of the sheet (Fig 1A).

#### Load cell

For the drilling procedure each sample was clamped on a load cell (Kistler, type 9257) with three charge amplifiers Fa Kistler no. 5007 (crosstalking between x/y/z typical <2%, max <5%) (Fig 1A). The load cell was fixed on a stiff table. The test specimen was placed 98 cm above the laboratory floor. The forces were recorded with a digital data acquisition system type HBM Spider8 and a laptop.

#### Drill bits, drilling machines and triaxial acceleration sensor

**Fig 2:**
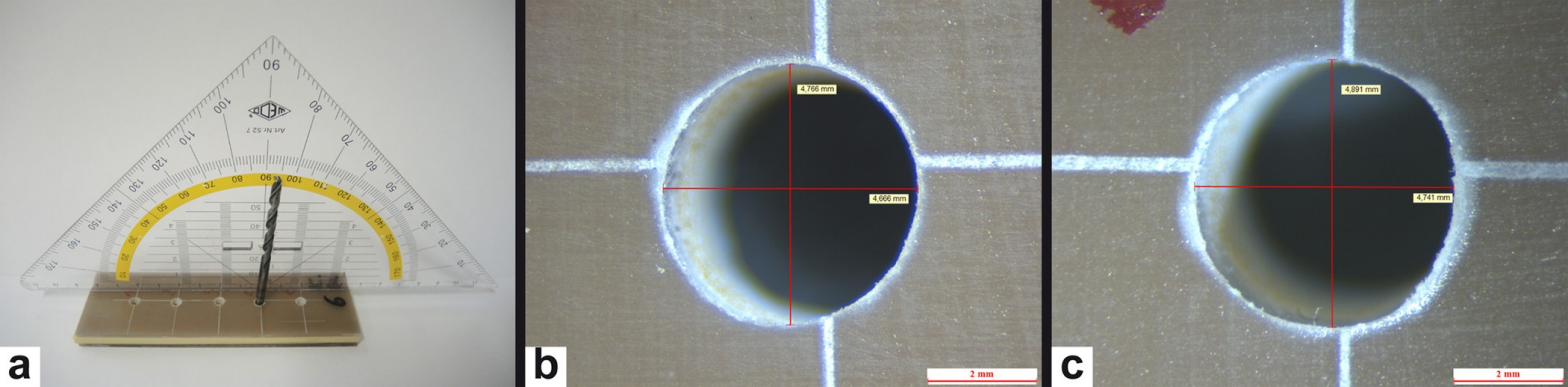
Resulting boreholes. (A) Measuring angle deviation with the help of a set square (B) Typical borehole electric drilling (C) Typical borehole manual drilling.

Fig 2B depicts the instrumented drilling machines. The electrical drilling machine used was the Type Osron produced by Kaiser Technology, with a pre-installed battery pack. The manual drill used was a traditional gear unit with crank handle. One turn of the crank generated two turns of the drill bit. Two equivalent drill bits with a diameter of 4.5 mm were used for both drilling machines. The maximal spindle speed was identified by analyzing the experimental data for the manual drilling and measured with a speedometer for the electrical device (Table 1).

**Table 1:** Spindle speed of drills.

Principal experiment	Drill diameter [mm]	Max. spindle speed [rpm]	Frequency [Hz]
Electric drill, test person 1	4.5	165	2.75
Electric drill, test person 2	4.5	160	2.,67
manual drill, test person 1	4.5	420	7
manual drill, test person 2	4.5	480	8

Triaxial acceleration sensors, model PCB 356 A16 (Fig 1B, Fig 1C) were applied with glue to both drilling machines. Both sensors were connected to a coupler (type Kistler 5134) and measured the vertical (z) and horizontal (x,y) acceleration during the drilling process (Fig 1D). This data was also recorded with the same data acquisition system.

### Drilling procedure

Two neurosurgeons (AC first author and CB senior author) served as test persons and carried out the drilling. One of them was female with 6 years of neurosurgical experience (AC); the other was male with 18 years of neurosurgical experience (CB). Each test person produced 2 x 5 boreholes with the electrically driven drill (E1-E4), using one hand at the piston-handgrip, and 2 x 5 boreholes with the manual twist drill with the crank (E5-E8), and using two hands. After every series of 5 boreholes the test persons switched in order to minimize the bias induced by possibly decreasing muscle strength.

### Statistical analysis

During the drilling the following parameters were collected: completeness/incompleteness of material penetration by each single drilling procedure [%], duration of each drilling process [s], maximum drilling force [N], time average of drilling force [N] in all three directions x, y and z as well as maximum acceleration in the direction x,y and z. The accelerations in both horizontal plains (x and y) were vectorially summarized.

Impulse [Ns] is a vector quantity which expresses the drive and the direction of an object. It can also be described as the change in momentum which is equal to the integral of force with respect to time (J = ʃF dt) in the force direction. In this project the term “impulse” is used as an equivalent of the the forces applied over a period and allows the assessment of the overall exposure to the patient. Considering for example an equal force level, a long term force causes a higher impulse than a short term force.

The impulse in the x-, y- and z-direction and the total impulse were calculated from the measured force data over time as

$$\Delta \vec{p}(t)=\vec{J}(t)=\int_{0}^{t}\vec{F}(t)dt$$

and

$${\vec{J}}_{tot}={\vec{J}}_{x}+{\vec{J}}_{y}+{\vec{J}}_{z}$$

The motion of the drilling machines was measured with piezoelectric accelerometers. The two horizontal directions were added vectorially. Acceleration was expressed as absolute value from the maximum and the minimum data*│a-max│* [m/s^2^].

An ensemble of 5 drilling processes was used for averaging. The mean, the maximal and the minimal value of the 20 electrical and the 20 manual drilling processes were determined as well.

The constantly high frequent vibrations caused by the motor and the gearing inside the electrical drilling machine was suppressed during the analyzing process by a 30 Hz lowpass filter to allow better comparison between the manual and the electrical drill.

Eventually, the following variables were determined: the diameter [mm] of each hole was measured with the help of a ZEISS Axioscope 2 with Leica-Application-Suite-Software (Fig 2B, Fig 2C). The angular deviation [°] of each hole was estimated via a drill bit and a set square (Fig 2A).

## Results

### Subjective evaluation of the drilling processes

Subjectively, a quick decrease in arm muscle strength could be observed in the electrical drilling. Consequently, large force by the test person was needed to push the drill forward and to maintain the drilling progress, especially into the first layer (short fiber filled epoxy sheet) of the probes. The last layer (filled fiber sheet) of the sandwich structure was easier to penetrate because the load cell provided a hard abutment. For the case of manual drilling, it felt much easier to penetrate the artificial bone: Both hands were involved and it took a high frequency of drilling turns but subjectively less muscle force.

### Objective evaluation of the drilling processes

#### Parameters of the electrically driven drilling

The means of 4 x 5 drilling parameters are listed in Table 2. The drillings numbered 1–5 and 11–15 were carried out by AC, the drillings 6–10 and 16–20 were carried out by CB (Table 2, Table 3).

**Table 2:** Parameters of electrical drilling, Part 1 (E+no = mean of 5 drilling procedures).

Experiment no.	Penetration rate [%]	Drilling time [s]	Deviation in angles		Force–mean [N]**			Force–max [N]**		
			xz	yz	x	y	z	x	y	z
1–5 (E1)	60	58	1.7	1.4	2	0	60	7	6	84
6–10 (E2)	100	65	0.8	0.8	6	-5	68	12	9	86
11–15 (E3)	100	50	3.6	1,3	12	-5	98	21	15	132
16–20 (E4)	100	79	1.9	1.7	10	-9	77	19	18	123
max	100	163	5	4	18	5	116	25	22	142
mean	90	63	2	1.3	7	-5	76	15	12	106
min	69	26	0	0	-3	-13	44	3	3	65

**Table 3:** Parameters of electrical drilling, Part 2 (E+no = mean of 5 drilling procedures).

Experiment no.	Impulse (J = ʃF dt) [Ns]			Total impulse [Ns]	Acceleration │a-max│ [m/s^2^]**	
	x		y	z	p-tot*	xy***	z
1–5 (E1)	131	-6	3610	3616	4.7	0.4
6–10 (E2)	377.2	-309.3	4410.0	4438	3.7	0.8
11–15 (E3)	502.2	-213.7	4548.8	4591	6.4	0.6
16–20 (E4)	593.2	-526.5	4326.2	4399	5.7	0.8
max	1042	190	9159	9266	7.9	3.3
mean	434	-294	434	4574	5.1	0.8
min	-91	-939	-91	1525	2.4	0.1

** values with 30 Hz lowpass filter

*** vector sum

#### Parameters of manually driven drilling

The means of 4 x 5 drilling parameters are listed in Table 3. The drillings numbered 21–25 and 31–35 were carried out by AC, the drillings 26–30 and 36–40 were carried out by CB (Table 4, Table 5).

**Table 4:** Parameters of manual drilling, Part 1 (E+no = mean of 5 drilling procedures).

Experiment no.	Penetration rate [%]	Drilling time [s]	Deviation in angles		Force–mean [N]**			Force–max [N]**		
			xz	yz	x	y	z	x	y	z
21–25 (E5)	80	56	1,9	0,8	-0,6	11,6	33,9	9,3	21,5	58,8
26–30 (E6)	100	27	1,2	2,4	0,9	5,1	43,4	17,2	18,1	89,3
30–35 (E7)	80	54	0,9	0,2	-5,5	14,4	44,4	14,3	28,1	83,7
35–40 (E8)	80	24	0,3	1,1	-0,4	8,8	58,3	17	24,2	114
max	100	70	4,0	5	2	18	71	22	34	123
mean	80	38	1,1	1,1	-1,4	10	45	14,5	23	86,5
min	60	15	0,0	0,0	-11	3	30	7	16	52

**Table 5:** Parameters of manual drilling, Part 2 (E+no = mean of 5 drilling procedures).

Experiment no.	Impulse (J = ʃF dt) [Ns]			Total impulse [Ns]	Acceleration │a-max│ [m/s^2^]**	
	x		y	z	p-total*	xy***	z
21–25 (E5)	-9,7	523	1490,7	1583	13,8	2,2
26–30 (E6)	27,3	138,4	1158	1167	23,8	3,5
30–35 (E7)	-299,6	759,2	2352	2502	14,6	1,9
35–40 (E8)	-5,7	210	1312,2	1330	24,4	4,5
max	116	921	2758	2916	32,7	8,7
mean	-72	408	1578	1646	19,2	3,0
min	-647	73	930	933	9,9	1,4

** values with 30 Hz lowpass filter

*** vector amount

### Comparison of parameters between electrical and manual drilling

#### Effect on the penetration rate

The penetration rate was higher for the electrical drilling with a total average of 90% in comparison to the manual drilling with a total average of 80%.

#### Effect on the drilling time

**Fig 3:**
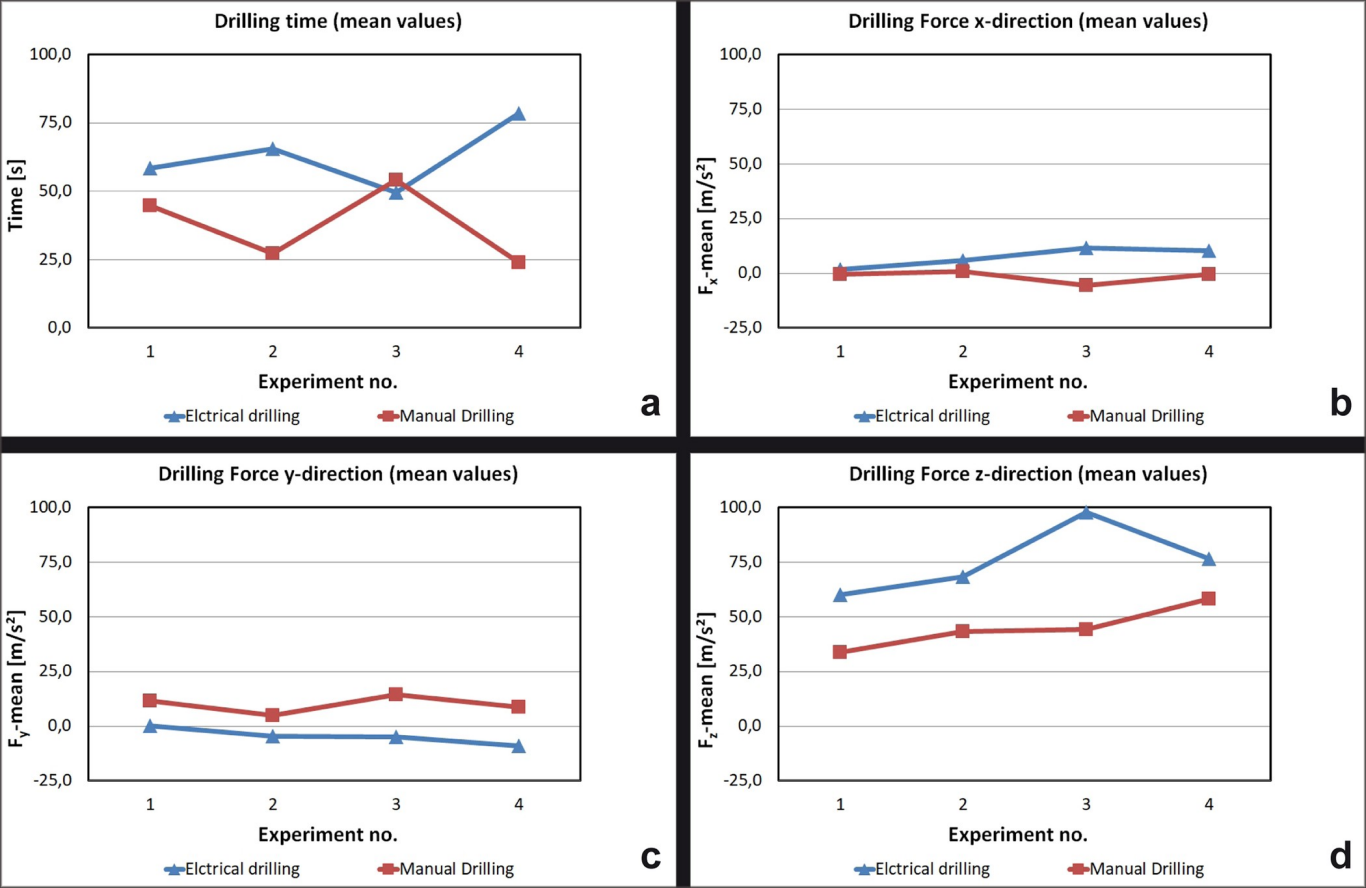
Drilling parameters (I). (A) Drilling time (B) Drilling Force x-direction (C) Drilling Force y-direction (D) Drilling Force z-Direction.

The mean time of producing a borehole was higher for the electrical drilling in comparison to the manual drilling (Fig 3A).

#### Effect on the horizontal forces F_x_ and F_y_ and the vertical force F_z_

Horizontally exerted forces could be measured during each drilling procedure. However, these values did not vary significantly between the electrical and the manual drilling. Even the forces in y-direction showed slightly higher levels in the case of manual drilling, caused by the rotation of the crank. A higher force in z-direction (“pressure”) was carried out by the operators in the electrical drilling **(Fig 3B, Fig 3C, Fig 3D)**.

#### Effect on the impulse

**Fig 4:**
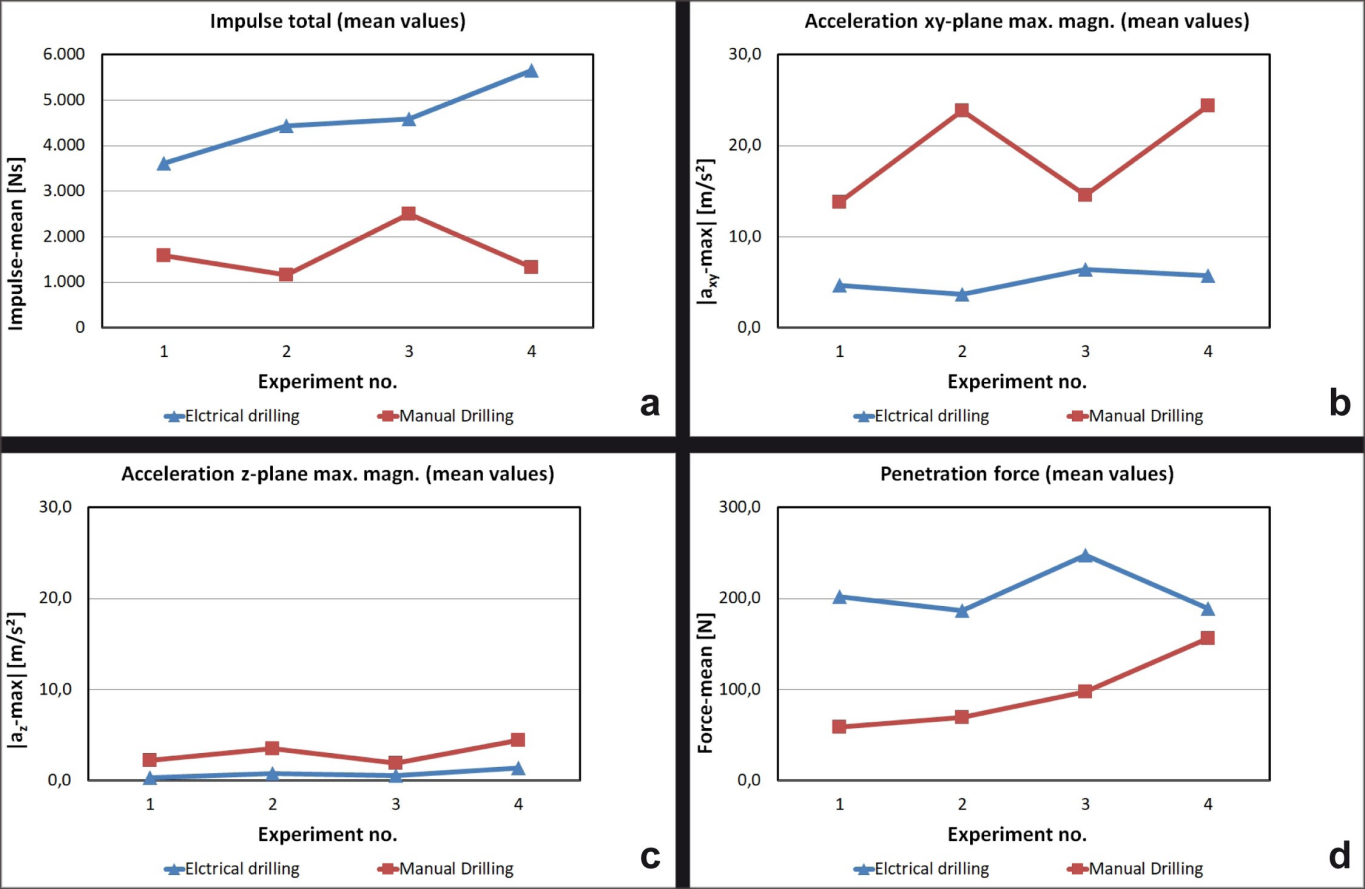
Drilling paramaters (II). (A) Impulse (B) Acceleration xy-plane (C) Acceleration z-plane (D) Penetration force.

Analogue to the higher vertical force z a higher impulse was needed in the electrical drilling to drive the machine through the bone sheet. It could also be observed that the impulse grows up with the number of drilled holes in the case of electrical drilling **(Fig 4A)**.

#### Effect on the acceleration

It could be observed that the rotation of the manual crank led to higher levels of acceleration in all directions. The manual drill was irregular, the curve showed oscillations, especially in the xy-plane. The electrical drill caused less oscillation. The curve was more steady-going **(Fig 4B, Fig 4C)**.

#### Effect on the penetration force

The penetration force correlates to the force z in Fig 3C: It was higher in the electrical drilling and represented the investment of pressure to maintain the drilling process **(Fig 4D)**.

### Comparison of the created boreholes

#### Hole diameter

**Fig 5:**
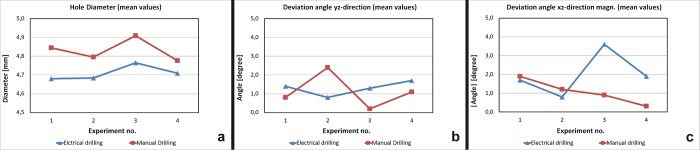
Borehole geometry. (A) Hole diameter (B) Deviation angle xy-direction (C) Deviation angle xz-direction.

The diameter of the drilling holes was measured under a microscope. On average, the electrical drilling holes showed a 1/10 mm smaller diameter than the manual drilling holes **(Fig 5A)**.

#### Angle deviation xz and yz

There are different observations concerning the angle deviation but a clear trend is difficult to determine. Fig 5B and 5C show that the angle deviation in the xz direction of the manual drilling decreases with the number of performed holes. The electrical drilling rather shows an increasing angle deviation in the xz and yz direction **(Fig 5B, Fig 5C)**.

## Discussion

### Subjective evaluation of the drilling process

The subjective evaluation of the drilling process lead to a surprising result: Both test persons stated that a higher muscular effort was needed for electrical drilling with the particular prototype machine utilized. One explanation for this detection is that the electric drilling machine could only produce a low spindle speed of 160 rpm. Recent research work [18] shows that the rotational speed has an effect on the force and torque in the sense that the force drops exponentially with the rotational speed. Reversely, by increasing the voltage, the spindle speed can be upgraded: By using 8 batteries instead of 6 our drilling machine runs with 215 rpm. However, an acceptable 325 rpm can only be generated with 14, 4 V—which comes up with 12 batteries. (Of course, the electric motor must be modified for this increased power.) Subsequently to our experiment, the used electric drilling machine and a second one of the same series were disassambled into their components. The frequency analysis (PSD) of the two electric drilling machines in the idle state pointed out that the behavior is not identical. Many excited frequencies are similar but the amplitudes are different and especially in the z-direction peaks at different frequencies could be observed. That leads to the assumption that the quality of manufacture needs improvement. Taken these two results the present electric drilling machine does not comply with the quality necessary for an easy, efficient, quick and trouble-free drilling in a neurosurgical emergency situation.

### Objective evaluation of the drilling process

The objective evaluation of the drilling process showed that the average vertical force z, the penetration force and the impulse were higher in the electric drilling. These confirm the subjective results described above. As a result of the low spindle speed, the drilling progress was generated by much more vertical pressure of the operating person. Likewise, a higher impulse was needed to drive the machine through the bone sheet. In contrast, the spindle speed of the manual drill with its maximum of 420 respectively 480 rpm required a lower input of pressure and impulse. It could be interesting to make corresponding measurements of vertical force and impulse with a trephine from the OR which has a higher rotational speed.

The penetration rate was higher in the electric drilling. Maybe the neurosurgeons could not control the manual drill as sensitive as the electric one because of the rotation of rge crank and rge correlated movements which could be observed in the higher level of acceleration in the xy- and z-direction (Fig 4B and 4C). A second reason may be the higher pressure and force, respectively, in z-direction, which is necessary to make the holes with the electric machine (Fig 3D).

### Geometry of the resulting boreholes

The microscopic analyses of the resulting 40 boreholes showed that the electric drill leads to more precise boreholes. In average, the holes had a smaller diameter. Furthermore, they were rounder and showed little irregularities in their margins at last in the beginning of the experiment (angle deviation yz). The effect abates in the course of the drilling series with less angle deviation even in the manual holes. This speaks for a certain training effect in turning the crank and conducting the drill simultaneously. But in summary, one can draw the conclusion that the electric drilling machine, in spite of the weak power and manufacturing quality, can be conducted and driven through the bone with less vibration and horizontal movement. The curves showed higher short time fluctuations in the manual drilling procedures.

### Limitations

Our experimental study has some limitations. Firstly, the biomechanical sheets do not include the curvature of the skull surface which holds the risk of slipping with the drill bit. Secondly, in a normal bedside or emergency room setting, there is no head fixation in a clamp and the operator has to cope with movements of the patients head. Thus, the plane sheets which were fixed into a clamping device depict a slightly idealized situation of drilling compared to real clinical practice. Thirdly, the electric drilling machine is a prototype product which is not applied in practice so far.

Our study neglects a comparison of the costs for the manual drill and the electric drill. But in the present case the cost for a one way and a re-sterilizable machine has to be considered.

## Conclusion

With regard to the emergency situation at the patient´s bedside, we can make the following statements: An electrically driven drilling machine contributes to a more controlled drilling in the sense that it generates less irregular movements in the xy direction and has a higher direct penetration rate. The round drilling holes nearly come up with the diameter of the drilling bit. This can help to achieve a precise trajectory for a ventricular catheter. In the manual drilling, the action for the operator is more complex: Turning the crank with the right and conducting the drilling machine with the left hand causes more lateral deviation of the drilling bit.

But the horizontal deviation and forces are only one limitation for a controlled drilling. Vertical forces and penetration forces, respectively, can have a negative impact on the result as well. If they are higher an increased risk of sudden penetration of the inner bone layer is the logical consequence. Those parameters change inversely to the rotational speed. The problem with the tested electric drill is its low rotational speed which requires higher vertical forces and impulse to maintain the drive of the drilling bit through the bone.

For neurosurgical emergency skull drilling, an electric drilling seems desirable because of the advantages identified in this study. However, it remains a challenge for manufacturers to combine the mechanical requirements with cost-efficiency and re-sterilizability. To the knowledge of the authors such drilling machines do not exist so far.

## Supporting information

S1 Table Single values of all drilling paramaters, electrical drilling (E 1–4). (XLSX)

S2 Table Single values of all drilling parameters, manual drilling (E 5–8). (XLSX)

## Abbreviations

OR: Operating Room
EVD: External Ventricular Drainage
n: Newton
s: seconds
a: acceleration
E: Experiment
t: time
